# RPL35A drives ovarian cancer progression by promoting the binding of YY1 to CTCF promoter

**DOI:** 10.1111/jcmm.18115

**Published:** 2024-03-04

**Authors:** Huijuan Wu, Liangbin Xia, Lu Sun, Dan Li, Xiangyu Liu, Hualin Song, Jindong Sheng, Ke Wang, Qinmei Feng

**Affiliations:** ^1^ Department of Gynecological Oncology Tianjin Medical University Cancer Institute and Hospital, National Clinical Research Center of Cancer, Key Laboratory of Cancer Prevention and Therapy Tianjin China; ^2^ Department of Obstetrics and Gynecology Renmin Hospital of Wuhan University Wuhan China; ^3^ Department of Gynecological Oncology Shanxi Province People's Hospital Shanxi China

**Keywords:** CTCF, mechanism, ovarian cancer, phenotypic, RPL35A

## Abstract

Ovarian cancer is one of the most common gynaecological malignancies with poor prognosis and lack of effective treatment. The improvement of the situation of ovarian cancer urgently requires the exploration of its molecular mechanism to develop more effective molecular targeted drugs. In this study, the role of human ribosomal protein l35a (RPL35A) in ovarian cancer was explored in vitro and in vivo. Our data identified that RPL35A expression was abnormally elevated in ovarian cancer. Clinically, high expression of RPL35A predicted short survival and poor TNM staging in patients with ovarian cancer. Functionally, RPL35A knock down inhibited ovarian cancer cell proliferation and migration, enhanced apoptosis, while overexpression had the opposite effect. Mechanically, RPL35A promoted the direct binding of transcription factor YY1 to CTCF in ovarian cancer cells. Consistently, RPL35A regulated ovarian cancer progression depending on CTCF in vitro and in vivo. Furthermore, RPL35A affected the proliferation and apoptosis of ovarian cancer cells through PPAR signalling pathway. In conclusion, RPL35A drove ovarian cancer progression by promoting the binding of YY1 and CTCF promoter, and inhibiting this process may be an effective strategy for targeted therapy of this disease.

## 1 INTRODUCTION

Ovarian cancer is a major gynaecological disease with a high mortality rate globally.^1^, ^2^ Recent statistics indicate that ovarian cancer has been the fifth most common cause of cancer‐related deaths in women over the past two decades.^1^, ^2^ It is often referred to as the ‘silent killer’ of women due to its hidden location and lack of effective treatment.^3^ The predominant method of treating ovarian cancer has been surgical resection, supplemented with chemotherapy and radiotherapy, but the overall outcomes have been unsatisfactory, with high rates of metastasis and recurrence.^4^ In recent years, with the advancement of our understanding of ovarian cancer at the molecular level, targeted therapy is being explored as a potentially effective treatment strategy, given its low toxicity advantages.^5^, ^6^ Various potential therapeutic targets have been identified for ovarian cancer, including tumour angiogenesis, signalling pathways, hormone receptors, homologous recombination deficiency and immune factors.^5^ Several drug targets have been used in clinical treatment, such as the anti‐vascular endothelial growth factor (anti‐VEGF) drug Bevacizumab,^7^ the poly (ADP‐ribose) polymerase (PARP) inhibitors like Rucaparib^8^, ^9^, ^10^ and the Mitogen‐activated protein kinase (MEK) inhibitor (VS‐6766) (11). Although the use of these targeted drugs effectively delays the progression of ovarian cancer and improves patient survival rates, long‐term use may lead to drug resistance.^11^, ^12^ Therefore, to improve the poor prognosis of ovarian cancer patients as much as possible, novel therapeutic targets need to be identified to develop more effective small‐molecule targeted drugs.

In addition to their role in ribosome assembly and protein translation, ribosomal proteins (RPs) have also been recognized for their significant functions that are not dependent on the ribosome.^13^ It has been reported that these RPs are involved in various physiological and pathological processes, such as triggering cell cycle arrest and apoptosis through activation of p53‐dependent or p53‐independent pathways in response to stress.^13^ Furthermore, the progression of cancer cells is often regulated by multiple tumour suppressors and oncogenic proteins, which control ribosomal biogenesis and protein synthesis.^14^ Therefore, insights into therapies targeting RPs offer new perspectives on the clinical implications of cancer therapy.^15^

RPL35A, also known as DBA5, L35A and eL33, is a constituent of the ribosome's large subunit, located at chromosome band 3q29‐qter.^16^ Analysis of gene expression in hepatocellular carcinoma (HCC) using a dense microarray of human cDNA showed increased expression of RPL35A.^17^ Additionally, RPL35A has been associated with the development of malignant brain tumours and may aid in identifying new targets for their diagnosis and treatment.^18^ Interestingly, RPL35A may also play a role in the cellular response to cytotoxic damage.^19^ However, there is currently insufficient evidence to support the notion that abnormal expression of RPL35A in cancer contributes to the progression of the disease. Therefore, our objective was to investigate the potential significant role of RPL35A in ovarian cancer, with the aim of establishing a theoretical foundation for targeted therapy in the treatment of this disease.

## 2 MATERIALS AND METHODS

### 2.1 Tissue microarray and immunohistochemical (IHC) staining

All procedures involving human experimental protocols were approved by the Committees of Tianjin Medical University Cancer Institute and Hospital. The tissue microarray was composed of tumour tissues (*n* = 107) and adjacent normal ovarian tissues (*n* = 8) of 107 clinical ovarian cancer patients. Subsequently, the tissue microarray was dewaxed with xylene for 15 min/time, hydrated with 100% ethanol for 10 min, rinsed with PBS and heated in citric acid buffer at 120°C for 20 min for antigen repair. After washing, the tissue microarray was incubated with the primary antibody (anti‐RPL35A, 1:50, Biorbyt, USA) at 4°C overnight, and incubated with the secondary antibody (Goat Anti‐Rabbit IgG H&L, Abcam, USA) at room temperature for 2 h. Finally, the tissue microarray was stained by diaminobenzene DAB for 10 min, washed by ddH_2_O, counterstained with haematoxylin, washed with PBS for 10 min, dehydrated and transparently sealed, and decolorized under microscope. Based on the combined scores of staining intensity and degree, the tissues were categorized as having either low RPL35A expression (below the median) or high RPL35A expression (above the median).

### 2.2 Cell culture

The ovarian cancer cell lines HO‐8910 (RRID: CVCL_6868), SK‐OV‐3 (RRID: CVCL_0532), OVCAR‐3 (RRID: CVCL_0465) and normal ovarian epithelial cells IOSE80 (RRID: CVCL_5546) were acquired from the Cell Resource Center based in Shanghai, China. All the cell lines were grown in a medium consisting of 90% DMEM and 10% FBS, and maintained at a temperature of 37°C in a humid environment with 5% CO_2_.

### 2.3 Target gene RNA interference lentiviral vector preparation and transfection

RPL35A‐targeting small hairpin RNA sequences (shRPL35A‐1: 5′‐GGTGTTTACGCCCGAGATGAA‐3′, 5′‐ACAGTCACTCCTGGCGGCAAA‐3′, 5′‐TTGGACACAGAATCCGAGTGA‐3′) and control Scramble sequences (shCtrl: 5′‐TTCTCCGAACGTGTCACGT‐3′) were synthesized and inserted into lentiviral vector with green fluorescent protein label, respectively. Lentiviral vectors containing targeted sequences (1 × 10^8^ TU/mL) and HO‐8910 and SK‐OV‐3 cells (2 × 10^5^ cell/mL) were mixed with lipofectamine 3000 (Invitrogen, USA) in DMEM with 10% FBS at 37°C for 30 min. The evaluation of the transfection effect was done by observing the expression of green fluorescent protein after a continuous 72 h culture. The selection of stable transfected cell lines was done using puromycin.

### 2.4 RNA extraction and quantitative real‐time PCR (qRT‐PCR)

Cell lines were treated with Trizol reagent (Sigma‐Aldrich, St. Louis, MO, USA) to isolate and purify total RNA following the manufacturer's instructions. Subsequently, cDNA was synthesized using the Promega M‐MLV kit (Vazyme, Nanjing, China). PCR reaction system (12 μL) containing cDNA, primers, SYBR Premix EX Taq (Vazyme) and RNase‐free H_2_O were prepared and qRT‐PCR detection was conducted in ABI StepOnePlus Real‐Time PCR System (Applied Biosystems, CA, USA). The 2^−∆∆CT^ method was used to calculate the relative expression of RNA, while GAPDH was utilized as an internal control. The primer sequences as follows: RPL35A forward primer 5′‐GAAGGTGTTTACGCCCGAGAT‐3′ and reverse primer 5′‐CGAGTTACTTTTCCCCAGATGAC‐3′; GAPDH forward primer 5′‐TGACTTCAACAGCGACACCCA‐3′ and reverse primer 5′‐CACCCTGTTGCTGTAGCCAAA‐3′.

### 2.5 Western blotting (WB)

Cell lines were lysed in ice‐cold lysis buffer (Cell Signal Technology) 15 min for protein extraction. Before subjected to 10% SDS‐PAGE, proteins were taking water bath at boiling water for 10 min. Then proteins were transferred to PVDF membranes, and the membranes were blocked with TBST solution (5% skimmed milk) for 1 h, then blots were incubated with primary antibodies (RPL35A, 1:2000, Abcam; GAPDH, 1:3000, Bioworld) overnight at 4°C, followed by incubated with HRP‐coupled secondary antibody (Goat Anti‐Rabbit, 1:3000, Beyotime). The enhanced chemiluminescence ECL + PlusTM western blotting detection system (Amersham Pharmacia Biotech, Arlington Heights, IL, USA) was used to observe immunoreactive proteins.

### 2.6 Celigo cell counting assay

The ovarian cancer cell lines HO‐8910 and SK‐OV‐3 were placed in 96‐well plates and kept at a temperature of 37°C for durations of 1, 2, 3, 4 and 5 days. At the same time each day, the Celigo® Image Cytometer (Nexcelom, Lawrence, MA, USA) was used to count the number of cells and plot proliferation curves to evaluate cell growth in each group.

### 2.7 Detection of cell apoptosis

HO‐8910 and SK‐OV‐3 cells were seeded in six‐well plates and incubated at 37°C for 7 days. After incubation, the cells were harvested, centrifuged at 1300 rpm and washed using 4°C D‐Hanks (pH = 7.2 ~ 7.4). The cells were then resuspended in 200 μL of 1 × binding buffer and 10 μL of Annexin V‐APC (eBioscience) was added for 15 min in the absence of light. FACScan (Millipore) was applied to assess the apoptotic rate.

### 2.8 Wound‐healing assay

HO‐8910 and SK‐OV‐3 cells were seeded in a 96‐well plate and incubated at 37°C until reaching 90% cell density. To initiate a scratching test, the cells were deprived of serum using serum‐free DMEM medium, resulting in the formation of a scratch on the plate. Photos were taken after adding 0.5% FBS. Subsequently, the cells were incubated in a 37°C, 5% CO_2_ incubator for 8 h, 24 h and 48 h, and photos were taken using a fluorescence microscope. The cell migration rate of each group was determined based on the images captured after the scratches.

### 2.9 Transwell migration assay

The upper chamber of the 24‐well cell culture plate was filled with HO‐8910 and SK‐OV‐3 cells, while 600 μL medium supplemented with 30% FBS was added to the lower chamber. After being incubated for 24 h, the cells were treated with 400 μL Giemsa for 5 min at room temperature and then examined using a microscope at a magnification of 200×.

### 2.10 Mouse xenograft model

The female BALB/C nude mice, aged 4 weeks, were obtained from Beijing Viton Lihua Laboratory Animal Technology Co., LTD. Following their acquisition, they were randomly assigned to four different groups, named empty vector (negative control), RPL35A (RPL35A overexpression), shCTCF (knocked down CTCF) and RPL35A + shCTCF (simultaneously upregulated RPL35A and downregulated CTCF). SK‐OV‐3 cells with interfered expression of RPL35A and CTCF were inoculated into nude mice by subcutaneous injection to establish xenograft tumour model. Ten days after the injection, tumour length and diameter were collected every 5 days, ensuring at least five measurements. The tumour volume was calculated according to the formula: *π*/6 × L × D × D, L represented the length and *D* represented the diameter. Cervical dislocation was used to kill the mice after 34 days, and to preserve them, their tumours were removed and weighed. Finally, tumour tissue was sliced for IHC staining to detect the expression of RPL35A (1:50, Biomol, A305‐106A), CTCF (1:400, Abcam, ab97080) and the proliferation marker KI67 (1:100, Abcam, ab16667).

### 2.11 Affymetrix microarray analysis

Shanghai Yibeirui Biomedical Science and Technology Co., Ltd. utilized RNA sequencing to detect gene expression in HO‐8910 cells. They used the Affymetrix human GeneChip PrimeView and scanned the outcomes with the Affymetrix Scanner 3000. Statistical significance of the raw data was assessed using a *t*‐test, and significance was determined with |Fold Change| ≥ 1.3 and FDR < 0.05. The data was further analysed using IPA, and a |*Z* – score| > 2 was considered to be significant.

### 2.12 Firefly luciferase & Renilla luciferase assay

Based on the promoter binding sites of YY1 and CTCF (chr16:67560526–67639,177), wild type (CTCF‐WT) and mutant CTCF (CTCF‐MUT) plasmids were constructed in this study (supplementary material). The above plasmids were transfected into HEK293T cells. The Promega Dual‐Luciferase system kit instructions were followed to perform the Firefly luciferase & Renilla luciferase assay. In brief, 75 μL of Dual‐Glo® Reagent was added to a 96‐well plate and allowed to sit at room temperature for 10 min. The luminescence value of the Firefly luciferase, which is the reporter gene, was then determined and recorded. Following this, 75 μL of Stop & Glo® Reagent was added and allowed to sit at room temperature for 10 min. The parameter value, which is the Renilla luciferase value, was determined and recorded.

### 2.13 Chromatin immunoprecipitation (CHIP)‐qRT‐PCR assay

The CHIP‐qRT‐PCR procedure was conducted following previously described methods.^20^ HO‐8910 and SK‐OV‐3 cells, either with overexpressed RPL35A or as negative controls (NC), were fixed with formaldehyde, lysed in SDS buffer and mechanically fragmented by sonication to break down the DNA. The protein‐DNA complexes were then precipitated using negative control (normal mouse IgG; Sigma, Cat. No. I5381), Histone H3 (D2B12) XP® Rabbit mAb (CST, Cat. No.4620) and anti‐YY1 (Proteintech, Cat. No. 66281‐1‐Ig) antibodies, respectively. The eluted DNA fragment was detected using specific primers for the CTCF promoter and SYBR premix (Vazyme) after separating the complex from the antibody. The primer sequence for CTCF as follows: 5′‐CCCAAGTTTATCACACCGCTCA‐3′ and 5′‐AAGGCAGCATCTAGGAAGTCATG −3′.

### 2.14 Statistical analysis

All the cell experiments in this study were repeated three times independently. The mean ± SD was used to express all the obtained data, which were then analysed using GraphPad Prism Version 8.0. The unpaired Student's *t*‐test and Fisher's exact test or Mann–Whitney *U* test were used to evaluate statistically significant differences between two groups, as appropriate. Differences with *p* values >0.05 were considered statistically significant.

## 3 RESULTS

### 3.1 RPL35A is highly expressed in human ovarian cancer

**FIGURE 1:**
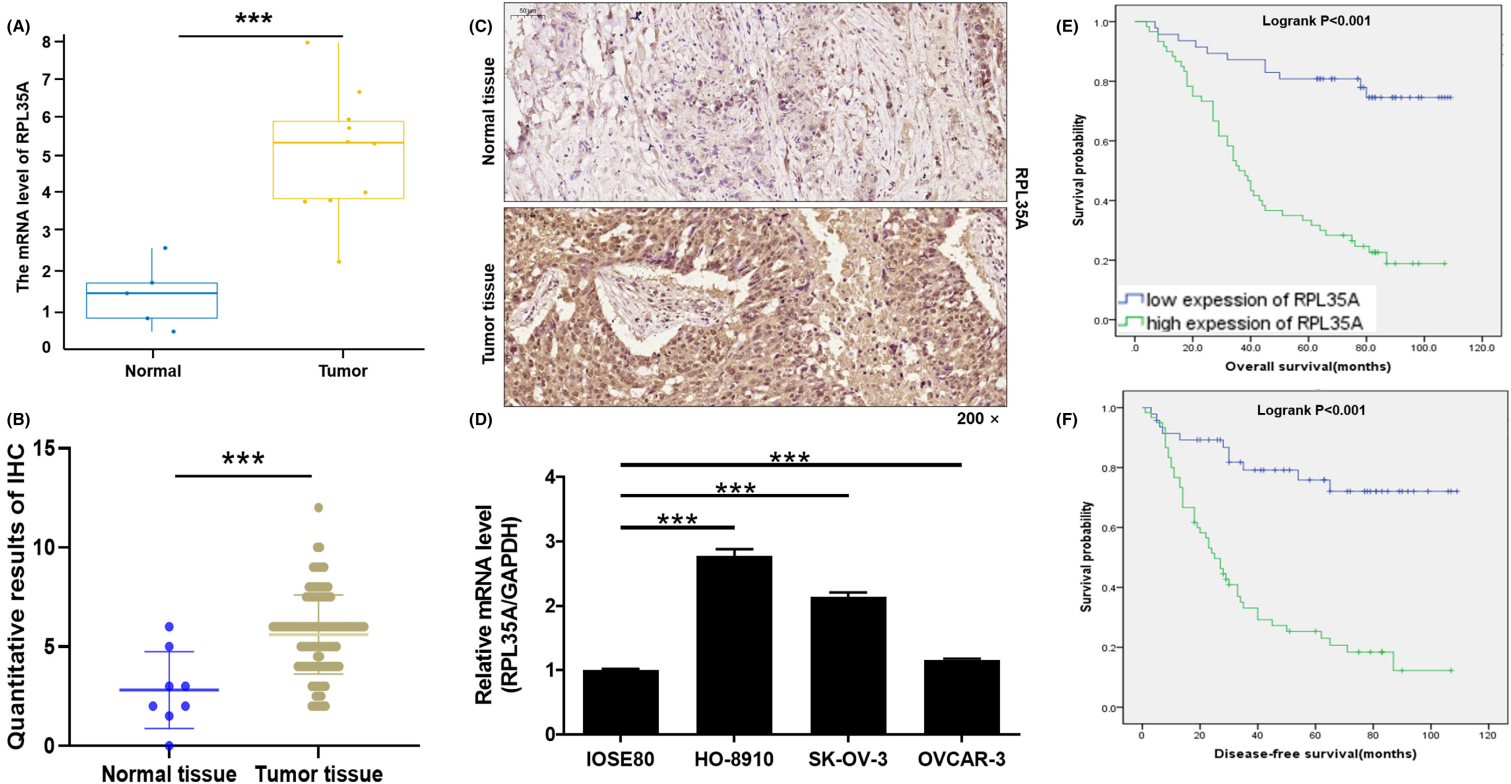
Correlation of RPL35A with clinicopathologic characteristics of human ovarian cancer. (A) The expression of RPL35A in tumour (*n* = 10) and normal (*n* = 5) was analysed from the Gene Expression Omnibus (GEO, GSE105437) data sets. (B) Expression patterns of RPL35A in ovarian cancer tissues (*n* = 107) and corresponding normal tissues (*n* = 8) was revealed by IHC staining. (C) Representative images of IHC in ovarian cancer tissues and corresponding normal tissues. (D) The expression of RPL35A in ovarian cancer cell lines (HO‐8910, SK‐OV‐3, OVCAR‐3) and normal ovarian epithelial cells (IOSE80) was evaluated by qRT‐PCR. ***p < 0.001. (E, F) Kaplan–Meier analysis based on medical records of patients with ovarian cancer showed the correlation between RPL35A expression and (E) overall survival and (F) disease‐free survival.

According to information from the Gene Expression Omnibus (GEO, GSE105437) data sets, it was discovered that the expression of RPL35A in tumour samples (*n* = 10) was significantly higher compared to normal samples (*n* = 5) with a *p* value less than 0.001, as shown in Figure 1A. In order to determine the clinical relevance of RPL35A in human ovarian cancer, the expression patterns of RPL35A were examined in ovarian cancer tissues (*n* = 107) and corresponding normal tissues (*n* = 8) using IHC staining. Based on the IHC scoring results, a score of 6 or higher indicated high expression, while a score below 6 indicated low expression (*p* < 0.001; Figure 1B). High expression of RPL35A was observed in 60 out of 107 tumour tissues (56.1%) and in one out of eight adjacent normal tissues (12.5%; *p* < 0.001) (Table 1). The IHC images in Figure 1C showed that the expression of RPL35A in tumour tissues was higher compared to the corresponding normal tissues. Consistently, the expression of RPL35A in ovarian cancer cell lines (HO‐8910, SK‐OV‐3, OVCAR‐3) was significantly higher than that in normal ovarian epithelial cells IOSE80, especially in HO‐8910 and SK‐OV‐3 cell lines (*p* < 0.001; Figure 1D).

**TABLE 1:** Expression patterns in ovarian cancer tissues and normal ovarian tissues was revealed by immunohistochemistry analysis.

RPL35A expression	Tumour tissue		Normal ovarian tissue		*p* Value
	Cases	Percentage	Cases	Percentage	
Low	47	43.9%	7	87.5%	<0.001
High	60	56.1%	1	12.5%	

### 3.2 Correlation of RPL35A with clinicopathologic characteristics of human ovarian cancer

Furthermore, the relationship between RPL35A expression and clinicopathologic features in ovarian cancer patients, such as age, grade, stage, tumour size, T Infiltrate (T), lymphatic metastasis (N), metastasis (M) and recurrence, was analysed using the Mann–Whitney *U* test (Table 2). The results showed that RPL35A expression was significantly correlated with grade (*p* = 0.015), stage (*p* < 0.001), T (*p* = 0.001), N (*p* < 0.001), M (*p* = 0.004) and recurrence (*p* = 0.002). Furthermore, Spearman correlation analysis confirmed a significant positive correlation between high expression of RPL35A and tumour grade, stage, TNM and recurrence (Table 3). In addition, Kaplan–Meier was used to analyse the association between RPL35A expression level and survival of ovarian cancer patients. Analysis results from the Cancer Genome Atlas (TCGA) database showed that although there was no significant correlation between overall survival and RPL35A expression level, the survival of patients with high RPL35A expression was shorter than that of patients with low RPL35A expression, as depicted in Figure S1. Additionally, analysis of clinically collected medical records of ovarian cancer patients showed that high expression of RPL35A was associated with shorter overall survival and disease‐free survival (*p* < 0.001; Figure 1E,F). Based on these findings, it can be speculated that RPL35A may be associated with poor prognosis.

**TABLE 2:** Relationship between RPL35A expression and tumour characteristics in patients with ovarian cancer.

Tumor features	No. of patients	RPL35A expression		*p* Value
		Low	High	
All patients	107	47	60	
Age (years)				
<51	53	26	27	0.331
≥51	53	21	32	
Grade				
I	8	5	3	0.015
II	10	7	3	
III	70	24	46	
Stage				
1	6	5	1	<0.001
2	24	16	8	
3	54	22	32	
4	23	4	19	
Tumour size				
<12.8 cm	50	26	24	0.117
≥12.8 cm	57	21	36	
T Infiltrate (T)				
T1	6	5	1	0.001
T2	24	16	8	
T3	77	26	51	
Lymphatic metastasis (N)				
N0	77	42	35	<0.001
N1	30	5	25	
Metastasis (M)				
M0	84	43	41	0.004
M1	23	4	19	
Recurrence of state				
No	22	16	6	0.002
Yes	85	31	54	

**TABLE 3:** Relationship between RPL35A expression and tumour characteristics in patients with ovarian cancer.

Tumor features	Index	RPL35A
Grade	Spearman correlation coefficient	0.260
	Significance (double tail)	0.014
	N	88
Stage	Spearman correlation coefficient	0.381
	Significance (double tail)	<0.001
	N	107
T Infiltrate (T)	Spearman correlation coefficient	0.334
	Significance (double tail)	<0.001
	N	107
Lymphatic metastasis (N)	Spearman correlation coefficient	0.343
	Significance (double tail)	<0.001
	N	107
Metastasis (M)	Spearman correlation coefficient	0.208
	Significance (double tail)	0.004
	N	107
Recurrence of state	Spearman correlation coefficient	0.295
	Significance (double tail)	0.002
	N	107

### 3.3 RPL35A promotes proliferation and inhibits apoptosis of ovarian cancer cells

**FIGURE 2:**
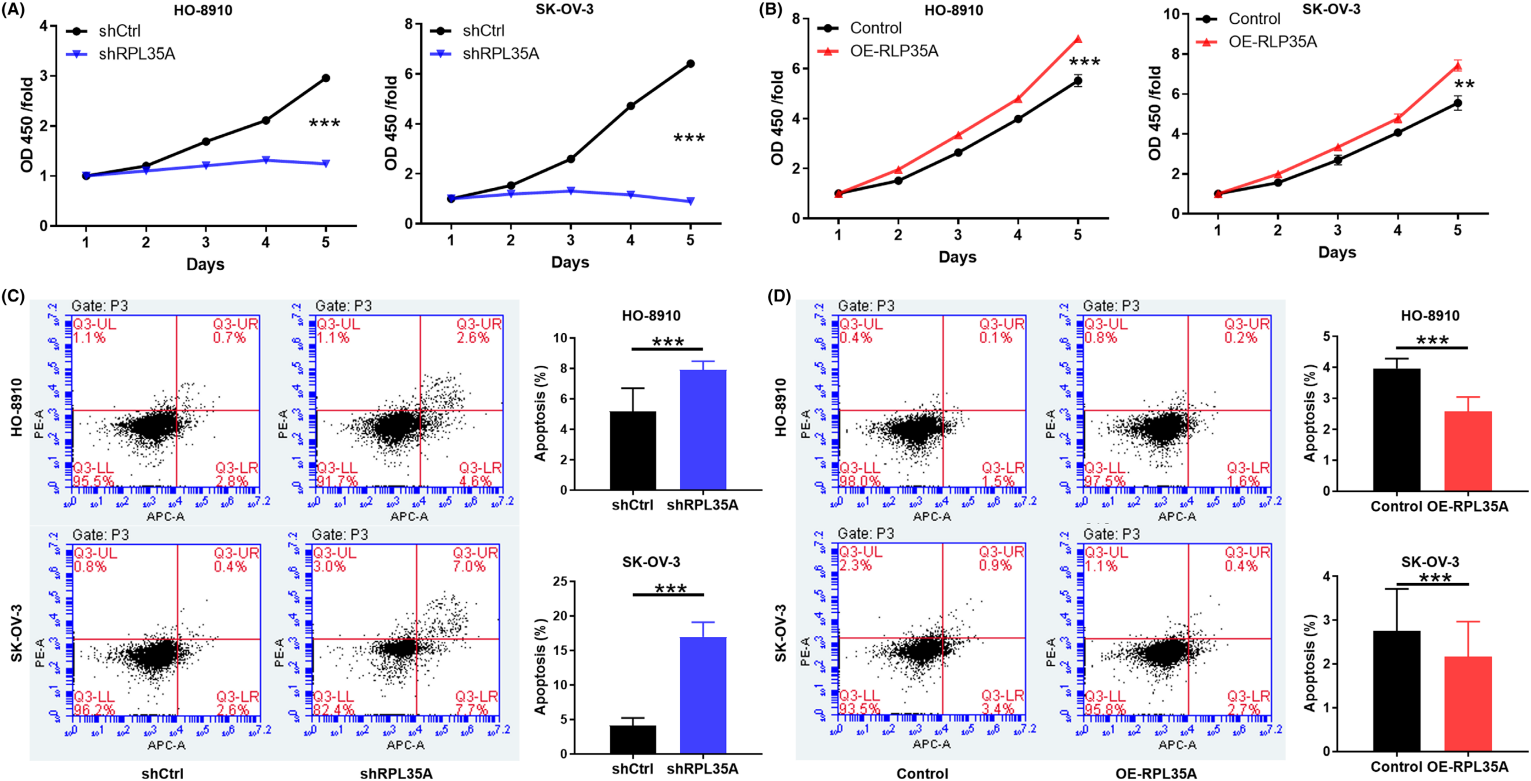
Effect of abnormal expression of RPL35A on proliferation and apoptosis of ovarian cancer cells. (A) The proliferation of HO‐8910 and SK‐OV‐3 cells was detected by Celigo counting assay after shCtrl and shRPL35A interference. (B) The proliferation of ovarian cancer cells overexpressed with RPL35A was detected by cell counting assay. (C, D) HO‐8910 and SK‐OV‐3 cells with (C) RPL35A knockdown and (D) RPL35A overexpression were evaluated by flow cytometry. All experimental data were independently repeated for 3 times to obtain the mean ± SD. **p < 0.01; ***p < 0.001.

To further evaluate the importance of RPL35A in ovarian cancer, we explored at the cellular level. Following that, the interference of shRNA (Figure S1) was used to disrupt the expression of RPL35A in HO‐8910 and SK‐OV‐3 cell lines, which was then measured using qRT‐PCR and WB. The results showed a significant reduction in the mRNA and protein levels of RPL35A in shRPL35A cells compared to shCtrl cells (*p* < 0.05; Figure S1). Similarly, RPL35A overexpressed HO‐8910 and SK‐OV‐3 cells were successfully constructed (Figure S1). Loss/gain‐of‐function experiments were conducted to reveal the role of RPL35A in the phenotypes of ovarian cancer. The data obtained from the cell counting assay indicated a decrease in the proliferative activity of ovarian cancer cells with RPL35A knockdown (*p* < 0.001; Figure 2A). In contrast, RPL35A‐overexpressed ovarian cancer cells showed stronger proliferation compared with control cells (*p* < 0.01; Figure 2B). Additionally, HO‐8910 and SK‐OV‐3 cells with RPL35A knockdown and RPL35A overexpression were evaluated by flow cytometry. As showed in Figure 2C,D, the apoptosis rate of HO‐8910 and SK‐OV‐3 cells with RPL35A knockdown was significantly higher than that of control cells, while that of ovarian cancer cells with overexpression of RPL35A was the opposite (*p* < 0.001). Collectively, RPL35A may promote proliferation and inhibit apoptosis of ovarian cancer cells.

### 3.4 RPL35A enhances the migration and invasion of ovarian cancer cells

**FIGURE 3:**
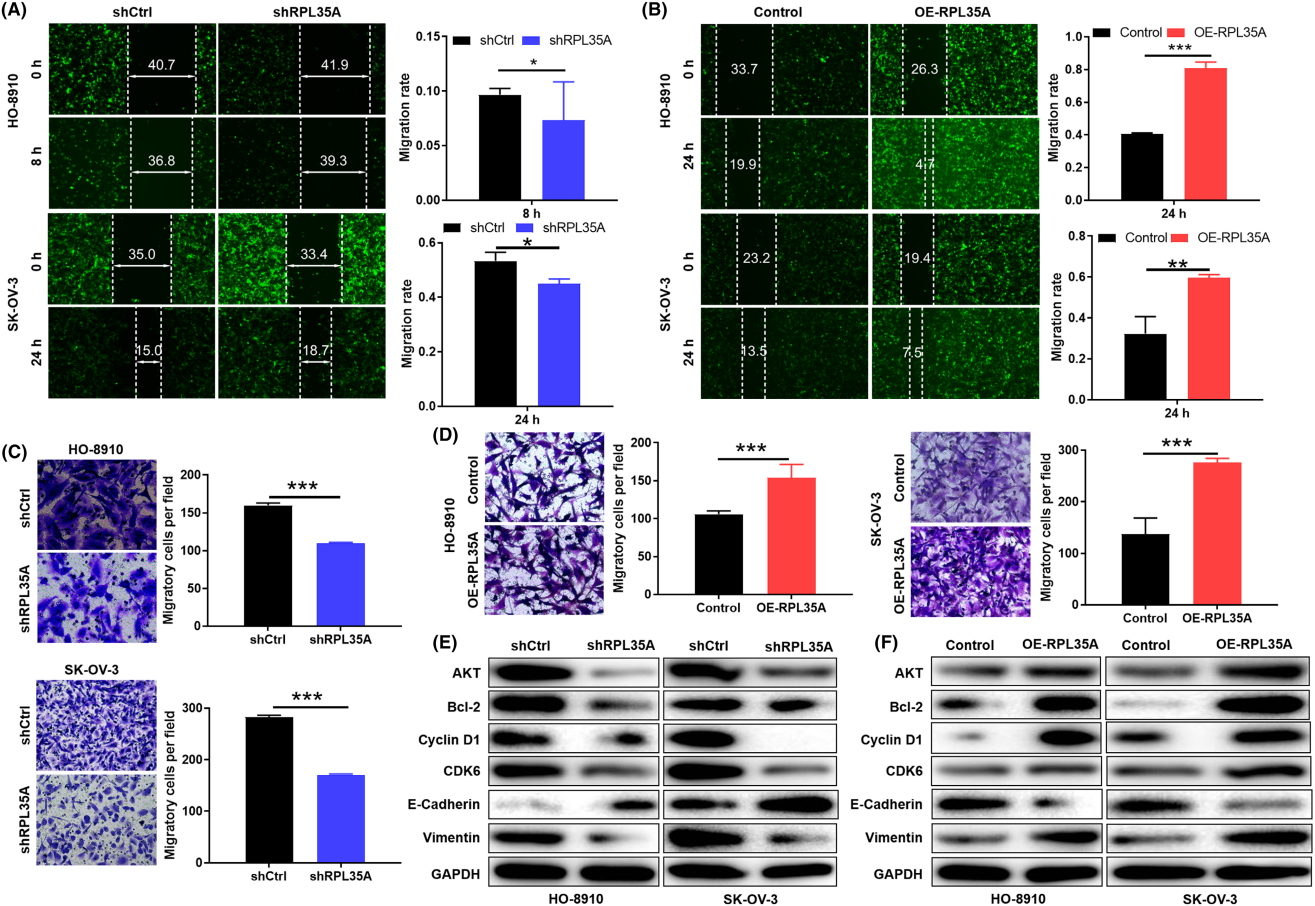
Effect of abnormal expression of RPL35A on migration of ovarian cancer cells. (A, B) HO‐8910 and SK‐OV‐3 cell migration after (A) RPL35A knockdown or (B) RPL35A overexpression was evaluated by wound‐healing experiments. (C, D) Transwell assay evaluated the invasion of HO‐8910 and SK‐OV‐3 cells after (C) shRPL35A interference or (D) RPL35A overexpression. (E, F) The expression levels of proteins related to phenotypes such as proliferation, apoptosis and EMT in the (E) RPL35A knocked down or (F) RPL35A overexpressed HO‐8910 and SK‐OV3 were detected by WB. All experimental data were independently repeated for 3 times to obtain the mean ± SD. *p < 0.05; ***p < 0.001.

Ovarian cancer cells with RPL35A knockdown and overexpression were evaluated for changes in their migration capacity by wound‐healing experiment and B Transwell tests. The wound‐healing assay demonstrated a suppressed cell migration ability in ovarian cancer cells when RPL35A was knocked down (*p* < 0.001; Figure 3A). As expected, ovarian cancer cells with overexpression of RPL35A showed enhanced migration (*p* < 0.001; Figure 3B). Moreover, the effect of RPL35A on ovarian cancer cell invasion was further confirmed through Transwell experiments, which showed a significant inhibition of invasion ability in shRPL35A HO‐8910 and SK‐OV‐3 cells compared to shCtrl cells (*p* < 0.05; Figure 3C). Overexpression of RPL35A showed the opposite result (*p* < 0.001; Figure 3D). In addition, the expression levels of proteins related to phenotypes such as proliferation, apoptosis and EMT in the RPL35A knocked down or RPL35A overexpressed HO‐8910 and SK‐OV3 were detected by WB. RPL35A knockdown downregulated AKT, Bcl‐2, Cyclin D1, CDK6 and Vimentin, upregulated E‐cadherin, while RPL35A overexpression showed opposite trend (Figure 3E,F). Overall, all these in vitro experiments provided evidence of the critical inhibitory effect of RPL35A knockdown in the malignant progression of ovarian cancer cells.

### 3.5 RPL35A promotes the direct binding of transcription factor YY1 to CTCF in ovarian cancer cells

**FIGURE 4:**
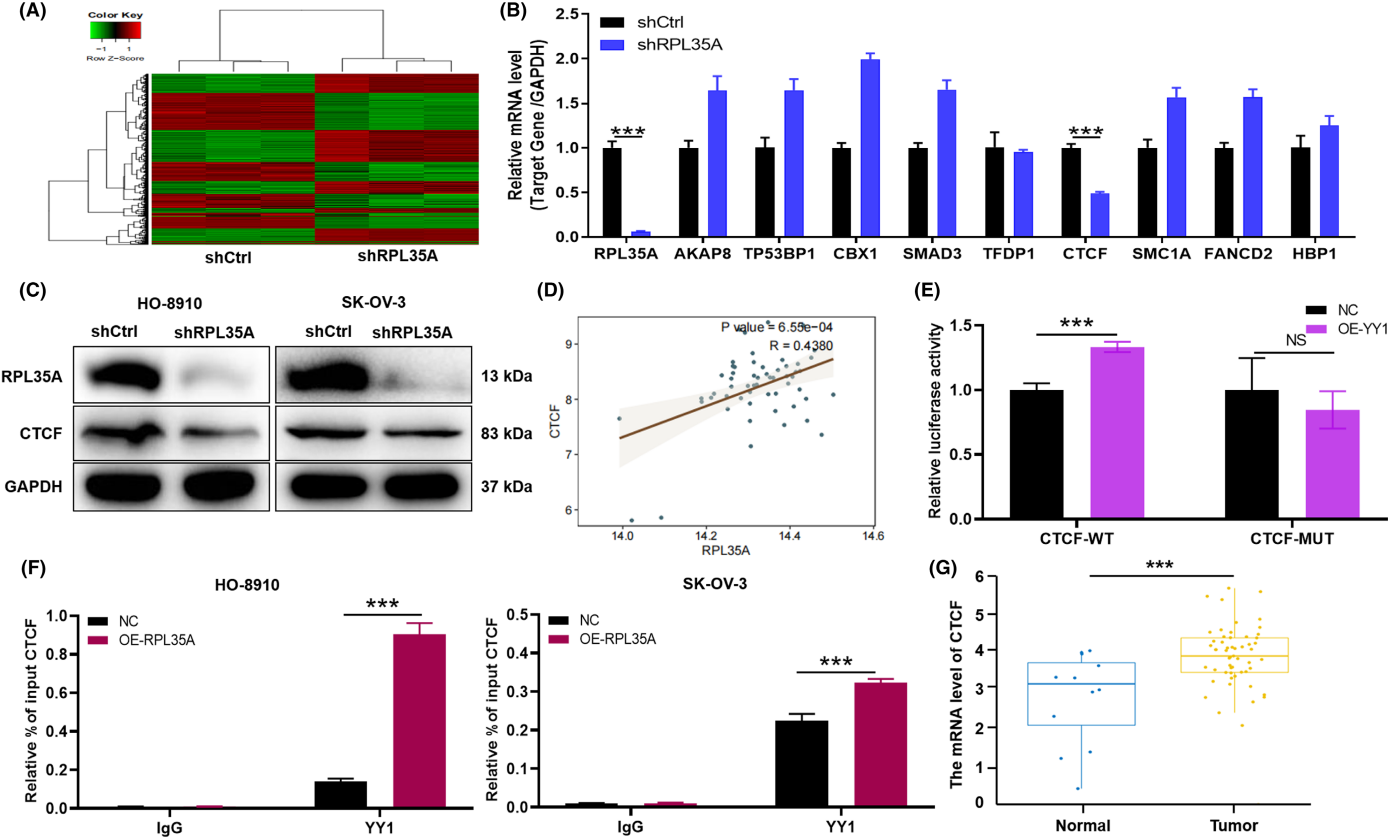
RPL35A promotes the direct binding of transcription factor YY1 to CTCF in ovarian cancer cells. (A) Downstream differentially expressed genes were identified by RNA sequencing between shRPL35A and shCtrl ovarian cancer cells. (B) Some genes with the most significant multiples of difference were selected and verified again by qRT‐PCR. (C) CTCF expression was evaluated by WB in HO‐8910 and SK‐OV‐3 cell after shCtrl and shRPL35A interference. (D) Pearson correlation analysis revealed a significant positive correlation between RPL35A and CTCF expression. (E) Wild type (CTCF‐WT) and mutant CTCF (CTCF‐MUT) plasmids were transfected into HEK293T cells and performed Firefly luciferase & Renilla luciferase assay. (F) CHIP assay showed the binding of YY1 to CTCF promotor. Chromatins were isolated from HO‐8910 and SK‐OV‐3 cells with RPL35A overexpression, and specific primers for CTCF promotor was used to DNA quantification. The enrichment percentage = 2% × 2[CT (input sample) − CT (IP sample)]. Normal IgG and histone H3 were used as negative and positive controls, respectively. (G) The expression of CTCF in tumour and normal was analysed from the GEO data sets. All experimental data were independently repeated for three times. ***p < 0.001.

In order to reveal the molecular mechanism of RPL35A regulating the progression of ovarian cancer, the following researches were carried out. Firstly, downstream differentially expressed genes were identified by RNA sequencing between shRPL35A and shCtrl ovarian cancer cells. Here, 2283 upregulated genes and 2188 downregulated genes were identified according to the screening criteria |Fold Change| ≥ 1.3 and FDR <0.05 (Figure 4A). Subsequently, some genes with the most significant multiples of difference were selected and verified again by qRT‐PCR. Among these genes, RPL35A knockdown led to the most significant down‐regulation of CTCF (*p* < 0.001; Figure 4B). However, there was no significant difference in the expression levels of other genes between shRPL35A and shCtrl (Figure 4B). Consistently, the results of WB confirmed the above phenomenon again (Figure 4C). It is interesting to note that the analysis of Pearson correlation showed a noteworthy positive correlation between the expression of RPL35A and CTCF (*p* < 0.001, *R* = 0.438; Figure 4D).

To determine the molecular mechanism between RPL35A and CTCF, the following investigation were conducted. YY1 is known to be a widely expressed transcription factor that plays a role in enhancer‐promoter structural interactions, similar to how CTCF‐mediated DNA interactions occur.^21^ Based on the promoter binding sites of YY1 and CTCF (chr16:67560526‐67639177), wild type (CTCF‐WT) and mutant CTCF (CTCF‐MUT) plasmids were constructed in this study. The above plasmids were transfected into HEK293T cells and Firefly luciferase & Renilla luciferase assay was performed. As illustrated in Figure 4E, YY1 could significantly enhance the expression of luciferase in CTCF‐WT but not in CTCF‐MUT (*p* < 0.001), suggesting that YY1 and CTCF promoter regions did have direct binding effect. Moreover, CHIP‐qRT‐PCR showed that the relative input rate of CTCF in YY1 antibody group was significantly higher than that in IgG. In addition, compared to the control group, the relative input rate of CTCF was increased in HO‐8910 and SK‐OV‐3 cells due to the overexpression of RPL35A (*p* < 0.001; Figure 4F), suggesting that RPL35A could promote the direct binding of transcription factor YY1 to CTCF in ovarian cancer cells.

### 3.6 RPL35A regulates ovarian cancer progression depending on CTCF in vitro and in vivo

In addition, GEO database analysis showed that CTCF was highly expressed in ovarian cancer (*p* < 0.001; Figure 4G). Therefore, the mechanism of RPL35A and CTCF regulating ovarian cancer progression required to be further explored. In order to further understand the roles of RPL35A and CTCF in ovarian cancer cells, a sequence of assays involving loss/gain of their function were conducted both in vitro and in vivo. Firstly, we interfered with the expression of RPL35A and CTCF in ovarian cancer cells, and named empty vector (negative control), RPL35A (RPL35A overexpression), shCTCF (knocked down CTCF) and RPL35A + shCTCF (simultaneously upregulated RPL35A and downregulated CTCF).

**FIGURE 5:**
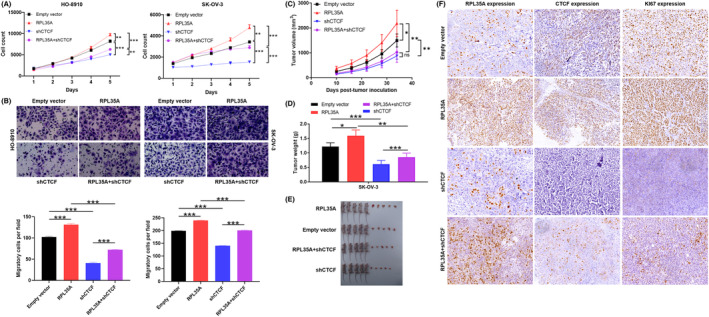
RPL35A regulates ovarian cancer progression depending on CTCF in vitro and in vivo. (A) The proliferation of HO‐8910 and SK‐OV‐3 cells was detected by Celigo counting assay. (B) Transwell assay evaluated the invasion of HO‐8910 and SK‐OV‐3 cells. (C–E) SK‐OV‐3 cells with interfered expression of RPL35A and CTCF were inoculated into nude mice by subcutaneous injection to establish xenograft tumour model and showed tumour volume and weight. (F) Tumour tissue was sliced for IHC staining to detect the expression of RPL35A, CTCF and the proliferation marker KI67. Empty vector as negative control, RPL35A as RPL35A overexpression, shCTCF as knocked down CTCF and RPL35A + shCTCF was simultaneously upregulated RPL35A and downregulated CTCF. Ns: no insignificance, *p < 0.05; **p < 0.01; ***p < 0.001.

CTCF‐knocked‐down HO‐8910 and SK‐OV‐3 cells showed a significant inhibition of proliferation and migration (*p* < 0.001; Figure 5A,B), which was consistent with the role of RPL35A knockdown in pancreatic cancer cells. In contrast, RPL35A overexpression showed a promoting effect on the progression of HO‐8910 and SK‐OV‐3 cells, including increased proliferation (*p* < 0.01) and enhanced migration (*p* < 0.001) (Figure 5A,B). Accordingly, RPL35A possessed a stimulative effect on human ovarian cancer cells. Furthermore, the promotion of malignant behaviours, such as enhanced proliferation and facilitated migration, in the RPL35A + shCTCF group was significantly higher compared to the shCTCF group, specifically observed in HO‐8910 and SK‐OV‐3 cells (*p* < 0.001; Figure 5A,B).

Ovarian cancer cells with interfered expression of RPL35A and CTCF were inoculated into nude mice by subcutaneous injection to establish xenograft tumour model. Over a period of time, differences in tumour volume and weight were observed. As illustrated in Figure 5C–E, compared with the control group, tumour size of RPL35A overexpression group was the largest (*p* < 0.05), while CTCF knockdown group was the smallest (*p* < 0.01). Interestingly, knockdown of CTCF partially reversed the promotion of tumour growth by RPL35A overexpression (*p* < 0.01). IHC staining experiments consistently confirmed that, CTCF knockdown partially reversed the regulation of RPL35A overexpression on the proliferation marker KI67 in tumour tissues (Figure 5F). Collectively, RPL35A regulated ovarian cancer progression through CTCF in vitro and in vivo.

### 3.7 RPL35A regulates ovarian cancer progression through PPAR signalling pathway

**FIGURE 6:**
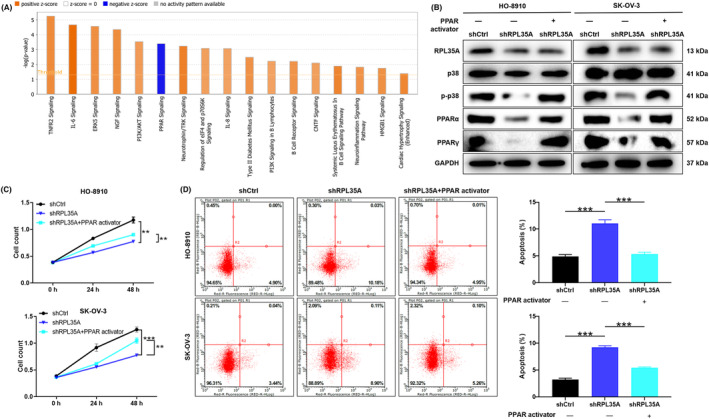
RPL35A regulates ovarian cancer progression through PPAR signalling pathway. (A) Ingenuity pathway analysis (IPA) results showed that down‐regulation of RPL35A resulted in significant enrichment of multiple signalling pathways. (B) The expression of typical components of PPAR signalling pathway was evaluated by WB in HO‐8910 and SK‐OV‐3 cells with RPL35A knockdowns were treated with PPARγ activator (Troglitazone, 10 μM). (C) After treated with PPARγ activator, proliferation of HO‐8910 and SK‐OV‐3 cells was detected by Celigo counting assay. (D) Apoptosis rates of HO‐8910 and SK‐OV‐3 cells were evaluated by flow cytometry after treated with PPARγ activator. **p < 0.01; ***p < 0.001.

Ingenuity pathway analysis (IPA) results showed that down‐regulation of RPL35A resulted in significant enrichment of multiple signalling pathways, including inhibition of the PPAR signalling pathway (Figure 6A). By WB assay, RPL35A knockdown in HO‐8910 and SK‐OV‐3 cells inhibits the PPAR signalling pathway, including the phosphorylation of p38, PPARα and PPARγ protein expression (Figure 6B). Furthermore, the expression of these typical components was partially reversed when HO‐8910 and SK‐OV‐3 cells with RPL35A knockdowns were treated with PPARγ activator (Troglitazone, 10 μM) (Figure 6B). Similarly, proliferation and apoptosis of HO‐8910 and SK‐OV‐3 cells with RPL35A knockdowns were partially alleviated when treated with Troglitazone drugs (*p* < 0.01; Figure 6C,D). Therefore, these results suggested that RPL35A may affect ovarian cancer progression through PPAR signalling pathway.

## 4 DISCUSSION

Ovarian cancer is currently the fifth leading cause of cancer‐related death in women, approximately 1.4 million women worldwide die from it each year.^22^ The improvement of the situation of ovarian cancer urgently requires the exploration of its molecular mechanism to develop more effective molecular targeted drugs. Of note, there is documented evidence suggesting that the ribosome protein RPL35A is linked to the advancement of both brain tumours and gastric cancer, implying that it could potentially serve as a point of focus for both diagnosis and treatment.^18^, ^23^ This study has explored and revealed the role of RPL35A in ovarian cancer, considering the information provided above. Firstly, we identified RPL35A expression in ovarian cancer based on databases and clinical tissue samples. As expected, RPL35A expression was abnormally elevated in ovarian cancer. Moreover, correlation of RPL35A with clinicopathologic characteristics of human ovarian cancer was analysed. High expression of RPL35A in patients with ovarian cancer was clinically associated with short survival and poor TNM staging, suggesting its potential as a diagnostic and prognostic marker for this disease. Furthermore, through cytofunctional validation, we found that RPL35A knockdown resulted in decreased proliferation, migration and enhanced apoptosis of ovarian cancer cells. Therefore, these results suggested that RPL35A may be a driver of ovarian cancer progression.

Of course, only the results of functional verification are not enough. In order to clarify our conclusions, this study conducted exploration of the molecular mechanism. Firstly, downstream differentially expressed genes were identified by RNA sequencing between shRPL35A and shCtrl ovarian cancer cells. After screening and verification, we found that RPL35A was positively correlated with CTCC‐binding factor (CTCF) expression. Previous study had demonstrated that CTCF plays a key role in organizing chromatin into TAD structures but it can also function as a transcription factor.^24^ Such small molecules can alter gene regulation and contribute to some of the underlying mechanisms of oncogenic transcription programs.^24^ Moreover, the ubiquitously expressed transcription factor YY1 contributes to enhancer‐promoter structural interactions in a manner analogous to DNA interactions mediated by CTCF.^21^ The current study conducted Firefly luciferase & Renilla luciferase and CHIP assays, which revealed that overexpression of RPL35A facilitated the direct interaction between transcription factor YY1 and CTCF in ovarian cancer cells.

CTCF, a transcription factor containing 11 zinc fingers (ZFs), has been reported to have involvement in various cancers including breast cancer, hepatocellular carcinoma, lung cancer and prostate cancer.^25^, ^26^, ^27^, ^28^ Results involving CTCF regulation of cancer progression may be attributed to gene transcriptional activation as well as transcriptional inhibition. In a prior study, it was reported that CTCF has the ability to inhibit p53 transcriptionally in breast cancer.^29^ Moreover, CTCF plays a carcinogenic role in neuroblastoma by activating MYCN or inhibiting tumour suppressors such as FOXD3.^30^, ^31^ CTCF interacts with telomeric repeat binding factor 2 (TRF2) to promote the proliferation of colorectal cancer.^32^ Functional tests were performed both in vitro and in vivo in this study to confirm the impact of RPL35A on the advancement of ovarian cancer cells through facilitating the direct interaction between YY1 and CTCF. Consistently, our data revealed that CTCF knockdown could partially reverse the regulation of RPL35A overexpression on ovarian cancer cells. Thus, we suggested that RPL35A regulated ovarian cancer progression depending on CTCF.

In the study, IPA results showed that down‐regulation of RPL35A resulted in significant enrichment of multiple signalling pathways, including inhibition of the PPAR signalling pathway. PPARs, which are nuclear receptors that serve as ligand‐activated transcription factors, consist of three isoforms namely PPARα, PPARβ/δ and PPARγ.^33^ The inhibition of the PPAR signalling pathway, including p38 phosphorylation and expression of PPARα and PPARγ proteins, occurs when RPL35A is knocked down in HO‐8910 and SK‐OV‐3 cells. Furthermore, the expression of these typical components was partially reversed when HO‐8910 and SK‐OV‐3 cells with RPL35A knockdowns were treated with PPARγ activator. Functionally, RPL35A affected the proliferation and apoptosis of ovarian cancer cells through PPAR signalling pathway. It is interesting to note that a previous study found that depletion of CTCF increased the amount of PPARγ in the nucleus.^34^ It should be mentioned that understanding the molecular mechanism linking CTCF and PPARγ is the main limitation of this study, and will be the main focus of our future research.

In ovarian cancer, our data shows that the expression of RPL35A is abnormally high. This high expression of RPL35A is clinically associated with short survival and poor TNM staging in ovarian cancer patients. Functionally, RPL35A knock down inhibits ovarian cancer cell proliferation and migration, enhances apoptosis, while overexpression has the opposite effect. More specifically, RPL35A promotes the direct binding of the transcription factor YY1 to CTCF in ovarian cancer cells. In summary, our findings suggest that the progression of ovarian cancer is driven by RPL35A through the promotion of the YY1‐CTCF binding, and targeting this process could be an effective therapeutic approach for this disease.

## AUTHOR CONTRIBUTIONS

**Huijuan Wu:** Conceptualization (equal); writing – original draft (equal). **Liangbin Xia:** Conceptualization (equal); writing – review and editing (equal). **Lu Sun:** Data curation (equal). **Dan Li:** Data curation (equal). **Xiangyu Liu:** Data curation (equal). **Hualin Song:** Data curation (equal). **Jindong Sheng:** Formal analysis (equal). **Ke Wang:** Formal analysis (equal). **Qinmei Feng:** Conceptualization (equal).

## FUNDING INFORMATION

Tianjin Key Medical Discipline (Specialty) Construction Project (TJYXZDXK‐009A).

## CONFLICT OF INTEREST STATEMENT

The authors do not have any financial or non‐financial interests to disclose that are relevant.

## Supporting information


Figure S1.

## Data Availability

The data produced during this study can be found in both the article and its supplementary data files.
