# Supplementary figures and images for: RPL35A drives ovarian cancer progression by promoting the binding of YY1 to CTCF promoter
Source: J Cell Mol Med. 2024 Mar 4;28(6):e18115. doi: 10.1111/jcmm.18115 (PMC10910871; doi:10.1111/jcmm.18115)

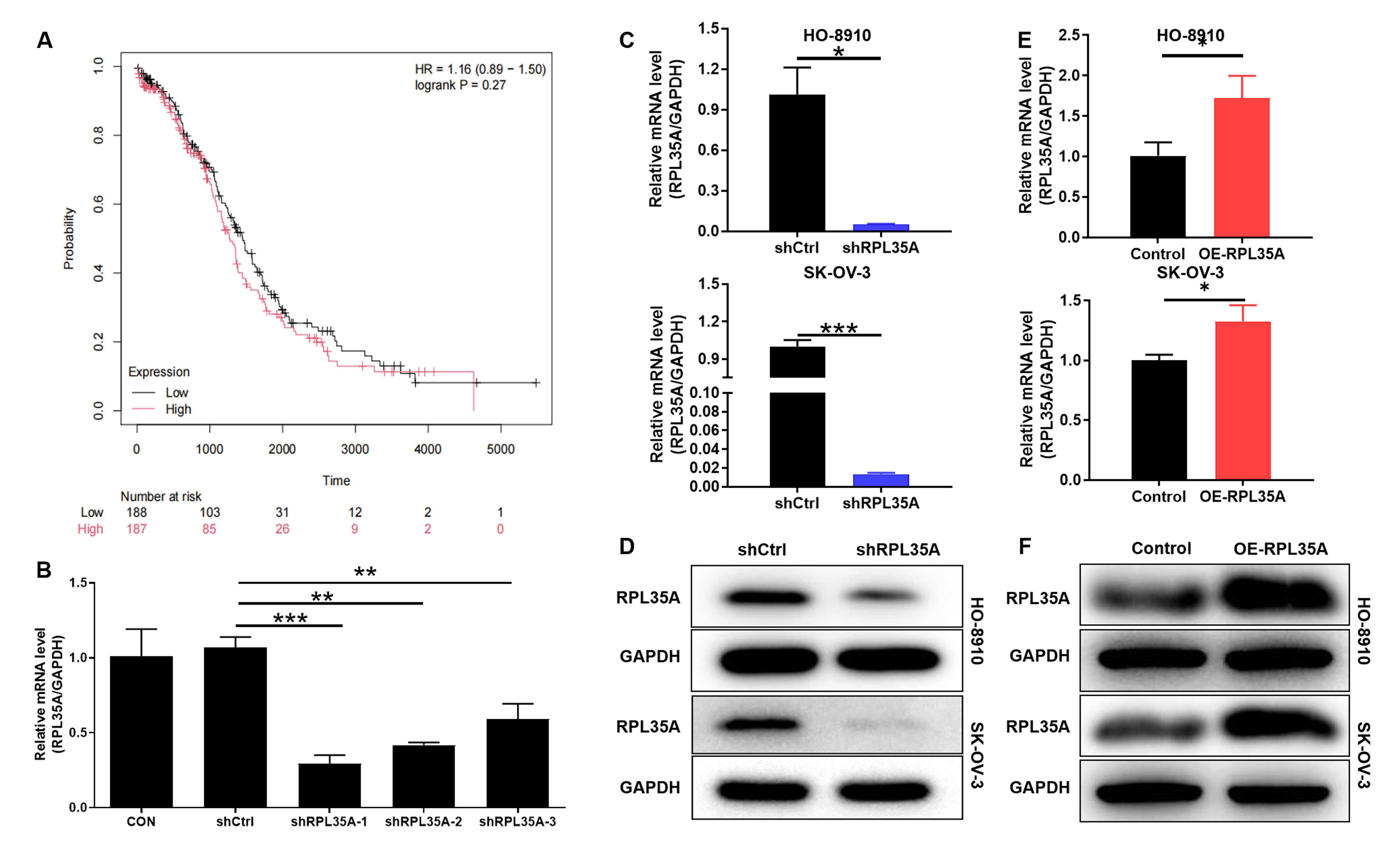

Supplement: Supplementary file 1 — Figure S1. [file JCMM-28-e18115-s001.tif]
